# Generation and Modulation of Controllable Multi-Focus Array Based on Phase Segmentation

**DOI:** 10.3390/mi13101677

**Published:** 2022-10-05

**Authors:** Zihan Liu, Jiaqing Hou, Yu Zhang, Tong Wen, Lianbin Fan, Chen Zhang, Kaige Wang, Jintao Bai

**Affiliations:** 1 State Key Laboratory of Photon-Technology in Western China Energy, International Collaborative Center on Photoelectric Technology and Nano Functional Materials, Key Laboratory of Optoelectronics Technology in Shaanxi Province, Institute of Photonics & Photon Technology, Northwest University, Xi’an 710069, China; 2 USTC Shanghai Institute for Advanced Studies, Shanghai 201315, China; 3 The 404 Company Limited China National Nuclear Corporation, Jiayuguan 735100, China

**Keywords:** phase segmentation, multi-focus array, modulation factor, numerical simulation, parallelized laser fabrication

## Abstract

A Circular-Sectorial Phase Segmentation (CSPS) noniterative method for effectively generating and manipulating muti-focus array (MFA) was proposed in this work. The theoretical model of the CSPS was built up based on vectorial diffraction integral and the phase modulation factor was deduced with inverse fast Fourier transform. By segmenting the entrance pupil into specified regions, which were sequentially assigned with the values carried out by phase modulation factor, the methodology could generate flexible MFAs with desired position and morphology. Subsequently, the CSPS was investigated in parallelized fabrication with a laser direct writing system. The positioning accuracy was greater than 96% and the morphologic consistency of the parallelly fabricated results was greater than 92%.

## 1. Introduction

The multi-focus array (MFA) is significant to modern optical techniques as it improves the parallel processing capacity of the optical systems, such as direct laser writing system [1,2], fluorescence microscope [3,4], and optical tweezers [5,6].

By means of beam splitter [7,8,9], microlens array [10,11,12], and interferometers [13,14], people could obtain diverse of multiple focus arrays. Meanwhile, the pattern of the arrays was changeless and the focal spots were specifically settled. At present, the liquid crystal on silicon (LCoS) device [15,16,17], which could realize dynamic manipulation of optical parameters for each pixel in beam transverse, has been preferred by more and more researchers. A variety of MFAs with flexible manipulation capacity of each focus could be obtained by loading appropriate phase maps on the LCoS.

There are several approaches to produce LCoS phase diagrams for MFAs, such as fractional Talbot effect phase-only modulation (FTEPM), Gerchberg–Saxton (GS) algorithm, weighted Gerchberg–Saxton (WGS) algorithm, and iterative Fourier transform algorithm (IFTA). In the work of Lin, the multifocal spot arrays with different number and spacing were obtained by modulating the fractional Talbot parameter. It was also capable of generating non-Airy arrays by combining the spatial structured phase with the fractional Talbot phase [18]. Based on the GS algorithm, Nogrette demonstrated the implementation of two-dimensional arrays that could contain up to 100 spots with arbitrary geometries for atom trapping [19]. In view of the vectorial property of the focusing system, Shi proposed the vectorial iterative optimization GS algorithm based on vectorial Fourier transform, which could implement arbitrary MFAs with a higher quality and accuracy [20]. By introducing proportional feedback to accelerate convergence, Zhang carried out a 10 × 10 MFA with high consistency with WGS algorithm [21]. Meanwhile, Kim proposed the generation of large-scale MFAs by an optimized IFTA. With the identifying and removing of undesired phase rotation, the focal spots could achieve 103 with high uniformity [22].

However, the FTEPM lacks flexibility in dynamically manipulating focal spots of the array. Meanwhile, the iterative algorithm needs a large quantity of iterations in generating high-quality MFAs, which takes a lot of time and will produce multiple solutions as the quantity of focus increased. In recent years, people found that the noniterative algorithm paves a simple and efficient way of producing and manipulating the MFAs. For example, by designing a multi-zone plate, Zhu proposed a method that can produce MFAs with manipulable polarization orientation of each focal spot [23]. Mu demonstrated a type of phase plate for generating tighter MFAs from azimuthal polarization beams [24]. In a study by Guan, a dartboard phase filter was designed and combined with cylindrical vector beams to implement MFAs [25]. In our previous work, a strip segmentation phase was reported for flexibly implementing and manipulating MFAs in a high *NA* optical system [26]. 

Here, we proposed a noniterative method named Circular-Sectorial Phase Segmentation (CSPS) for MFA generating and modulating. In the CSPS, the phase of the entrance pupil was equally segmented into a circular and a serial of sectorial regions, which were further divided into sub-regions. Subsequently, the phase diagrams for the MFAs were obtained by assigning these sub-regions with the phase values carried out by phase modulation factor. The performance of CSPS was studied with numerical simulation and validated in parallelized laser fabricating of micro-nano structures. The fabrication results indicated that the CSPS could produce controllable MFAs with good consistency.

## 2. Principles and Methods

### 2.1. The Principle of Focal Spot Positioning

**Figure 1:**
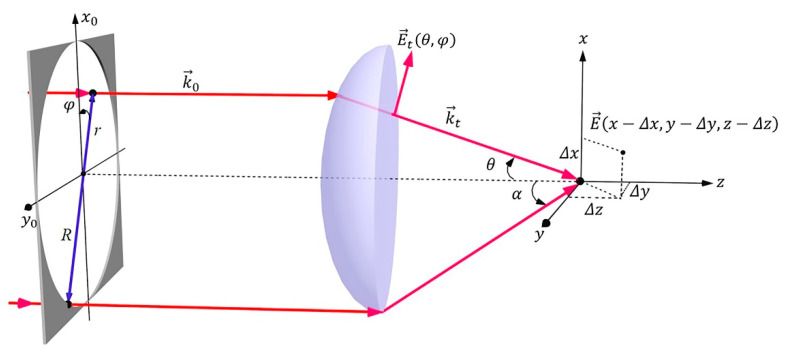
Schematic diagram of focusing beam by a high *NA* objective lens.

Figure 1 is the geometry for the tight focusing of the rays which are emitted from a point at infinity of the optical axis. The electric field intensity ***E*** at an arbitrary point near the focal plane can be described by the Debye diffraction integral as Equation (1). Here, for simplification, we omit the constant coefficients.

(1) $$E\left(x,y,z\right)=\int_{0}^{a}\int_{0}^{2\pi }P\left(\theta \right)E_{t}\left(\theta ,\varphi \right)\exp\left[i\left(k_{z}z-k_{x}x-k_{y}y\right)\right]\sin\theta d\varphi d\theta$$

where *P*(*θ*) is the apodization function of objective, ***E_t_*** (*θ*, *φ*) is the electric field of the transmitted light, *α* is the semi aperture angle of the objective lens, *θ* is the acute angle between the refracted ray and the optical axis, and *φ* is the azimuthal angle. *K_x_*, *k_y_*, and *k_z_* are the components of wave number in *x*, *y*, and *z* directions in the Cartesian co-ordinate system, respectively. The components can also be expressed as Equation (2) in the spherical co-ordinate system.

(2) $$\left\{ \begin{array}{l} k_{x}=-k\cos\varphi \sin\theta \\ k_{y}=-k\sin\varphi \sin\theta \\ k_{z}=k\cos\theta \end{array}\right.$$

Therefore, Equation (1) can be rewritten as:

(3) $$E\left(x,y,z\right)=\int_{0}^{a}\int_{0}^{2\pi }P\left(\theta \right)E_{t}\left(\theta ,\varphi \right)\exp\left(ik_{z}z\right)/\cos\theta \cdot \exp\left[-i\left(k_{x}x+k_{y}y\right)\right]dk_{x}dk_{y}$$

Equation (3) can be expressed as Equation (4) by using inverse Fourier transform:

(4) $$E\left(x,y,z\right)={ℱ}^{-1}\left\{P\left(\theta \right)E_{t}\left(\theta ,\varphi \right)\exp\left(ik_{z}z\right)/\cos\theta \right\}$$

According to the shift theorem of Fourier transform, the electric field is given by:

(5) $$E\left(x-\Delta x,y-\Delta y,z\right)={ℱ}^{-1}\left\{\exp\left[-i\left(k_{x}\Delta x+k_{y}\Delta y\right)\right]P(\theta )E_{t}(\theta ,\varphi )\cdot \exp\left(ik_{z}z\right)/\cos\theta \right\}$$

where the Δ*x* and Δ*y* are the displacement of the focal spot in *xy* plane. Let *Φ* = *k_x_*Δ*x* + *k_y_*Δ*y*, it is clear that the factor *Φ* determines the shifting of the focal spot. 

Suppose the *x*_0_, *y*_0_ are the Cartesian co-ordinates in the pupil plane, it has:

(6) $$\left\{ \begin{array}{l} x_{0}=r\cos\varphi \\ y_{0}=r\sin\varphi \end{array}\right.$$

where *r* is the radial position in polar co-ordinate in the pupil plane.

Considering the relationship:

(7) $$\sin\theta =rNA/RN_{t}$$

where *NA* is the numerical aperture of the objective lens and *N_t_* is the refractive index of immersion medium.

Therefore, the spatial frequency *k_x_* and *k_y_* can be alternately expressed as:

(8) $$\left\{ \begin{array}{l} k_{x}=-2\pi x_{0}NA/RN_{t}\lambda \\ k_{y}=-2\pi y_{0}NA/RN_{t}\lambda \end{array}\right.$$

Finally, after substituting *k_x_* and *k_y_* into phase function *Φ*, the phase modulator factor can be obtained as follows:

(9) $$\phi \left(x_{0},y_{0}\right)=\frac{2\pi }{\lambda }\left[\frac{NA}{RN_{t}}\left(x_{0}\Delta x+y_{0}\Delta y\right)\right]$$

### 2.2. The Principle of Phase Segmenting

In order to produce controllable MFAs, here we proposed a noniterative algorithm named Circular-Sectorial Phase Segmentation (CSPS) to produce the corresponding phase maps.

**Figure 2:**
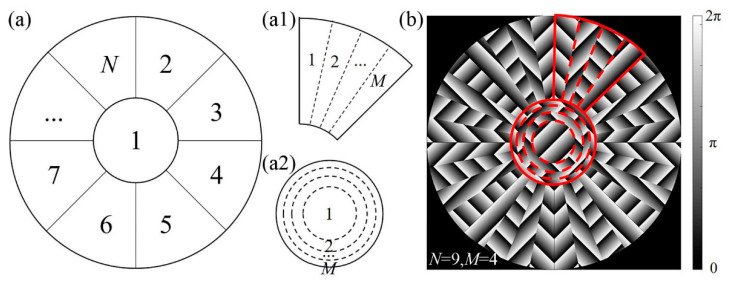
Schematic of phase segmentation in CSPS. (**a**) Geometry of segmentation regions in entrance pupil; (**a1**) segmenting sectorial region to sub-regions; (**a2**) segmenting circular region to sub-regions; (**b**) phase map for *N* = 9 and *M* = 4.

Assume the segmentation accuracy of the pupil is *N* and the focal spot quantity in the desired MFA is *M*. As shown in Figure 2a, the phase over the entrance pupil is segmented into a circular region and *N*–1 sectorial-shaped regions with the same size. Since the radius of the entrance pupil is *R*, the radius of the central circular region is $\frac{R}{\sqrt{N}}$ and the area of each divided region is $\frac{\pi R^{2}}{N}$. The parameter *N* impacts the quality of the focal spots. Theoretically, the focal spots of the array will gain better uniformity with sharper profiles as *N* increases. The upper limit of the *N* depends on the pixel resolution of the SLM.

As shown in Figure 2(a1), each sectorial-shaped region is equally divided into *M* sub-regions according to the desired number of focal spots. Meanwhile, the circular area in Figure 2(a2) is divided into *M* annular-shaped sub-regions as well. The radius of the annular sub-regions is r_1_, r_2_···r*_M_*, respectively. In addition, it has a relationship of r_1_:r_2_:···:r_*M*_ = 1:$\sqrt{2}$:···:$\sqrt{M}$.

Assume *n* is an integer from 1 to *N* and *m* is an integer from 1 to *M*. Then, the segmented sub-regions can be numbered as (*n*, *m*). For example, the *m*th annular-shaped sub-region in the central circular region is numbered as (1, *m*). For another example, the *m*th sectorial sub-region in the *n*th sectorial region will be numbered as (*n*, *m*). The rays in the *m*th sub-regions contribute to the *m*th focal spot of the MFA.

Finally, by assigning the numbered sub-regions with the values, which are carried out by Equation (9), the corresponding phase maps can be obtained. For instance, Figure 2b shows a phase map carried out with *N* = 9 and *M* = 4. The phase over the entrance pupil has been equally divided into a circular region and eight sectorial regions. The corresponding sectorial and circular regions are further divided into four sub-regions and filled with phase values.

## 3. Results and Discussion

### 3.1. Numerical Simulation

In the numerical simulation, the phase maps and light intensity distribution of the MFAs are carried out based on Matlab 2018b. The parameters in the simulation are consistent with those in the practical experimental systems. The light beam is Gaussian plane wave with wavelength *λ* at 800 nm. The entrance pupil radius *R* is 2.5 mm, the numerical aperture *NA* of objective lens is 1.4, and the refractive index n of image space is 1.518.

**Figure 3:**
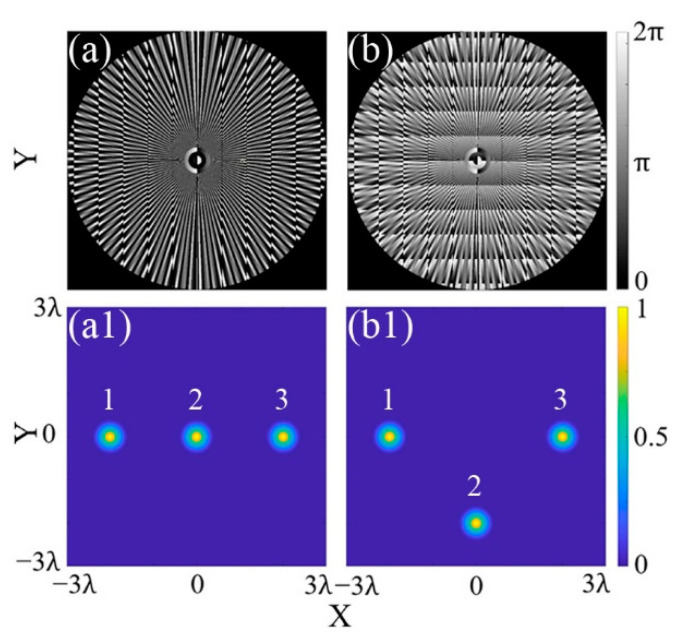
Generation and manipulation of a triple-focus array. (**a**,**a1**) Phase map and intensity distribution for a triple-focus array; (**b**,**b1**) phase map and intensity distribution for a triangular array.

For example, in Figure 3a, a triple-focus array (*M* = 3) is generated and manipulated based on the CSPS. In order to obtain an MFA with good uniformity, the accuracy parameter *N* is set to 100. According to Equation (9), the position of the triple focal spots depends on the values of (Δ*x*, Δ*y*). When the position parameter of the focal spots “1, 2, 3” is given as (Δ*x*, Δ*y*) = (−2λ, 0), (0, 0), (2λ, 0), the corresponding phase map will be carried out as in Figure 3a. The intensity distribution of the triple-focus array is shown as Figure 3(a1), in which the focus “2” is located at the origin, the focus “1” is located at (−2λ, 0), and the focus “3” is located at (2λ, 0), respectively. If the position parameter, (Δ*x*, Δ*y*), of the focus “2” is set as (0, −2λ), the phase map will be patterned as in Figure 3b. Meanwhile, the focus “2” will be moved to (0, −2λ), as shown in Figure 3(b1).

**Figure 4:**
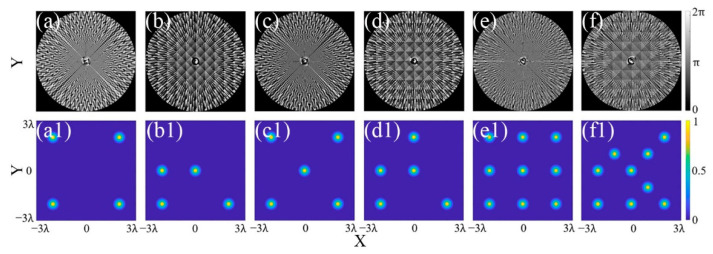
Phase maps and intensity distribution of different MFAs. (**a**,**b**,**a1**,**b1**) *M* = 4; (**c**,**d**,**c1**,**d1**) *M* = 5; (**e**,**f**,**e1**,**f1**) *M* = 9.

Further, the phase maps and corresponding intensity distribution for different MFAs are obtained by the CSPS. Setting *N* = 100, and *M* = 4, 5, and 9, respectively. Then, the phase maps for 4, 5, and 9 multi-focus arrays were obtained, as illustrated in Figure 4a–f. From Figure 4(a1–f1), it is seen that the multi-focus arrays with different shapes can be easily obtained by assigning different values to Δ*x* and Δ*y* of Equation (9). 

In addition, by substituting the phase expression of special beams into the phase modulation factor (9), the MFAs of special focal patterns can be obtained. For example, by substituting the Bessel beam phase factor *exp*{*ik*[(*h − rtanγ*) (*n* − 1)]} into Equation (9), the phase modulation factor will have the following expression:

(10) $$\phi \left(x_{0},y_{0}\right)=\frac{2\pi }{\lambda }\left[\frac{NA}{RN_{t}}\left(x_{0}\Delta x+y_{0}\Delta y\right)\right]+\left[\left(h-rtan\gamma \right)\left(N_{t}-1\right)\right]$$

where *γ* is the cone angle and *h* is the thickness of the cone lens. 

Similarly, the phase modulation factor for vortex focal spot array can be obtained by substituting the spiral phase factor *exp*(*ilφ*) into Equation (9):

(11) $$\phi \left(x_{0},y_{0}\right)=\frac{2\pi }{\lambda }\left[\frac{NA}{RN_{t}}\left(x_{0}\Delta x+y_{0}\Delta y\right)\right]+l\varphi$$

where *l* is the topological number. 

**Figure 5:**
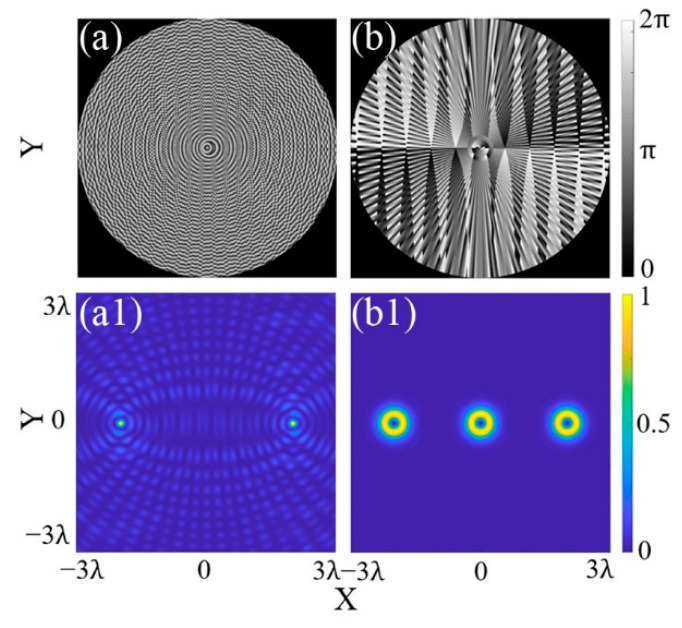
Phase maps and intensity distribution of specially structured MFA. (**a**,**a1**) Bessel light; (**b**,**b1**) vortex light.

Figure 5 shows the phase maps and light intensity distribution of specially structured MFAs based on the CSPS method. Figure 5a is the phase map for a Bessel light focus array based on Equation (10). The Bessel light array is obtained as Figure 5(a1) by setting *N* = 99, *M* = 2, and (Δ*x*, Δ*y*) = (−2λ, 0), (2λ, 0). The distances of the two Bessel light focal spots to the origin are −2λ and 2λ, respectively. 

Figure 5b is the phase map for a triple vortex focus array based on Equation (11). Letting *N* = 100, *M* = 3, and the topological charge *l* = 2. The position parameter of the triple focus is setting as (Δ*x*, Δ*y*) = (−2λ, 0), (0, 0), (2λ, 0), respectively. Then, the intensity distribution of the triple-focus array is obtained as shown in Figure 5(b1). The three vortex focal spots are distributed in the *X*-axis and their distances to the origin are −2λ, 0, and 2λ, respectively. 

**Figure 6:**
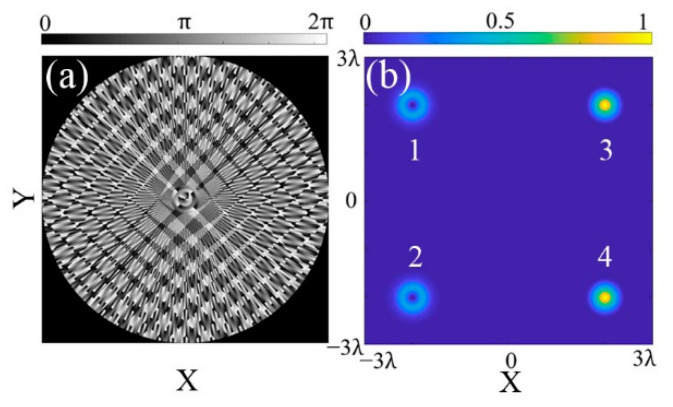
Morphology control of MFA. (**a**) Phase map; (**b**) intensity distribution of a vortex-Gaussian quaternion-focus array.

It is worth mentioning that the CSPS is capable of generating MFAs with different focal topographies as well. As shown in Figure 6, an MFA, which is composed by vortex focal spots (*l* = 2) and Gaussian light (*l* = 0), is obtained with *N* = 100, *M* = 4, and the phase modulation factor in Equation (11). The expected focal points are numbered as “1, 2, 3, 4” and the corresponding position parameter is set as (Δ*x*, Δ*y*) = (−2λ, 2λ), (−2λ, −2λ), (2λ, 2λ), (2λ, −2λ), respectively. Meanwhile, a conditional judgment parameter *t* is introduced to control the topography of the focal points. For example, if *t* is less than or equal to 2, the topological number *l* of focal point “1” and “2” will be set as 2 to generate the vortex focus. Otherwise, the topological number *l* will be set as 0 to generate Gaussian focus. Finally, a quaternion-focus array with different topological numbers is generated as shown in Figure 6b. These simulation results above indicate that the CSPS is efficient in generating multi-focus arrays with different spot quantity, shapes, and topography.

### 3.2. Analysis and Discussion

As explained in Section 2, in the CSPS, the entrance pupil is equally segmented into *N* regions, which are further divided into *M* smaller sub-regions, respectively. It is easy to find that the ray in one *M*th of the entire pupil should be the effective part for each focal spot of the MFA. Therefore, it should be noted that the more focal spots there are, the less pixels will be assigned for each focal spot. On the other hand, the factor *N* mainly impacts the distribution uniformity of these pixels for each focal spot, thereby having an influence on the appearance of the focal spots.

Take the LCoS (PLUTO-NIR-011, HOLOEYE) in the following experiment as an example. The pixel resolution (*P_s_*) of this LCoS chip is 1920 × 1080, the pixel size is 8 μm, and the liquid crystal panel size (*S*) is 15.36 mm × 8.64 mm = 132.7104 mm^2^. Considering the entrance pupil radius (*R*) of the microscope objective used in the experiment (PlanApo 100× *NA* 1.4, Olympus) is 2.5 mm, the effective modulation region of the spatial light modulator (*S*′) is π*R*^2^ ≈ 19.625 mm^2^. Therefore, the effective pixel number (*P_e_*) in the experiment equals to *P_s_* × (*S*′/*S*) ≈ 306,640; therefore, the effective pixels for each focus in MFA are 306,640/*M*. 

According to the work of Zhu [26], the width of an annular sub-region should reach at least two pixels to realize the modulation of a focal spot in the multi-focus array. Therefore, it has:

(12) $$\left\{ \begin{array}{l} r^{\prime }=\frac{R}{\sqrt{N}}=r^{\prime \prime }+2u\\ m=\frac{\pi \left(r^{\prime }{}^{2}-r^{\prime \prime }{}^{2}\right)}{u^{2}}\\ M=\frac{P_{e}}{Nm} \end{array}\right.$$

where *r*′ and *r*″ are the radii for the *M*th and (*M* − 1)th ring of the circular sub-region, respectively. *U* is the pixel size of the spatial light modulator; *m* is the quantity of pixels in the sub-regions. The size the MFA generated by CSPS depends on *P_e_*, *N*, and *m*.

### 3.3. Experimental Study

Here, by employing the CSPS method into a two-photon laser direct writing system, the reliability of the CSPS in generating controllable multi-focus arrays is further investigated. 

**Figure 7:**
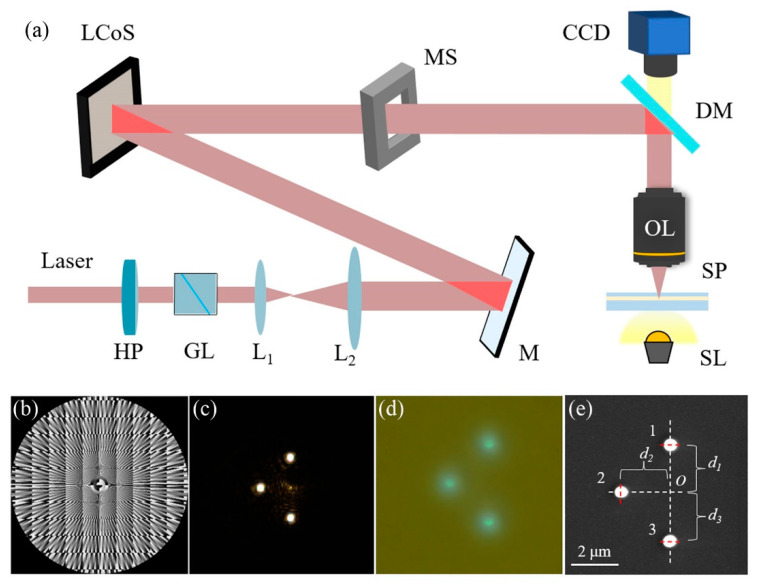
Parallel fabrication of micro-nano structures based on CSPS. (**a**) Schematic of experiment set up; (**b**) phase map for a triple-focus array; (**c**) CCD image of laser array in focal plane; (**d**) exposure of photoresist with laser array; (**e**) SEM image of fabrication results.

The setup for the multi-focus parallel fabrication is shown in Figure 7a. The laser light source is a picosecond laser with a center wavelength at 775 nm (KWKATANA-08 HP, NKT Photonics). HP and GP, respectively, refer to half-wave plates and Glan prisms, which are combined to adjust the laser power in the system. L_1_ and L_2_ are the lens for beam expanding and M is a silver mirror. The corresponding phase map for the MFA will be uploaded on the LCoS (PLUTO-NIR-011, HOLOEYE) during the experiment. MS is a mechanical shutter (SH05, Thorlabs) for exposure time control. DM is a dichroic mirror DM (Semrock, Di02-R532/800-t2-25×36) settled in front of the objective OL (PlanApo 100× *NA* 1.4, Olympus). SP is the sample for laser fabricating and SL is the safety light for illuminating. The image on the focal plane will be shot by the CCD camera (MER-U3-L, Daheng Optoelectronics). 

The sample is prepared with a photoresist, ethoxylated bisphenol A diacrylate (Sartomer) monomer with 0.5 wt% photoinitiator DETC (7-diethylamino-3-thenoylcoumarin) (J&K). A glass wafer is applied to prevent the OL from the pollution of the sample. 

For an example, a triple-focus array is generated and applied in the fabrication. As shown in Figure 7b, the phase map for the triple-focus array is generated by the CSPS with (Δ*x*, Δ*y*) = (0, 2 μm), (−2 μm, 0), (0, −2 μm). The resolution of the phase map is consistent with the spatial light modulator pixel. The outer diameter of the effective modulation area is 5 mm to match the diameter of the entrance pupil. By uploading the phase map to the LCoS, the triple-focus array spot pattern in the OL plane can be observed as Figure 7c. It is seen that a weak zero-order light is settled in the central area; however, it can be ignored, for it is too weak to initiate polymerization. The zero-order spot can be eliminated by adding a blazed grating to the phase map as well. 

In the fabrication, the laser power at the entrance pupil is set to 130 mW and the exposure time is 10 s to initiate the polymerization. The parallel fabricating process is shown in Figure 7d. The DETC photoresist emits green fluorescence under the irradiation of 775 nm laser. After the exposure, the sample is developed in isopropanol for 10 min and rinsed by deionized water. Figure 7e is the SEM image of the obtained micro-nano structure. Assume *d*_1_, *d*_2_, and *d*_3_ are the distance of point *O* to the structure “1”, “2”, and “3”. It has *d*_1_ = 2.017 μm, *d*_2_ = 2.069 μm, and *d*_3_ = 2.087 μm, which is highly consistent with the expected parameter. In addition, the diameters of the structures “1~3” are 554 nm, 542 nm, and 565 nm, respectively. It indicates that the focal light intensity and morphology of the triple-focus array are highly consistent with each other.

**Figure 8:**
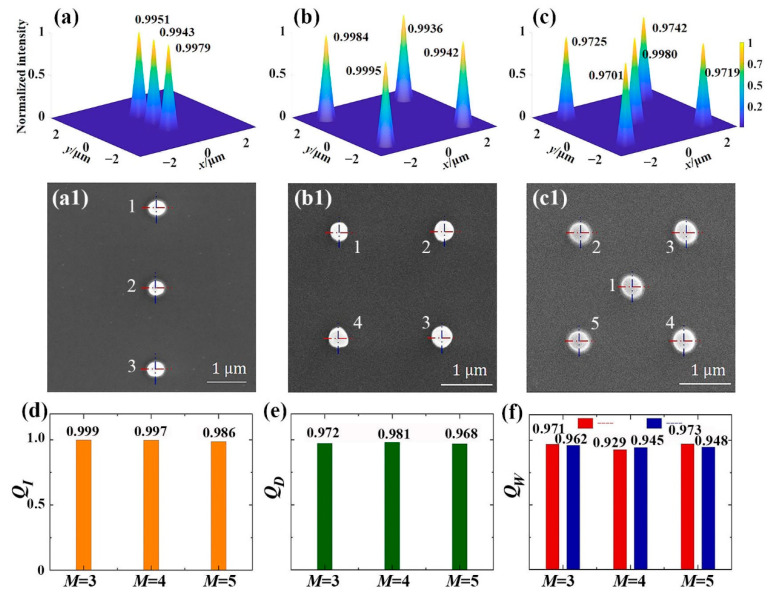
Parallel fabrication of micro-nano structure array. intensity distribution of (**a**) triplet-focus, (**b**) quaternion-focus, and (**c**) quintuplet-focus array generated by CSPS; parallel fabrication results of (**a1**) triplet-focus, (**b1**) quaternion-focus, and (**c1**) quintuplet-focus array; analysis of (**d**) intensity distribution in numerical simulation, (**e**) positioning accuracy in fabrication, and (**f**) morphological consistency of micro-nano structures.

Figure 8 shows the parallel fabrication of micro-nano structures based on CSPS. The intensity distribution of MFAs generated by the CSPS with *N* = 100 and *M* = 3, 4, and 5 has been revealed as Figure 8a–c. It indicates that the intensity distribution of the focal spots is highly consistent with each other, theoretically. 

The position parameter in the triple MFA is given as (Δ*x*, Δ*y*) = (0, 2 μm), (0, 0), (0, −2 μm), setting (Δ*x*, Δ*y*) = (−1 μm, 1 μm), (1 μm, 1 μm), (1 μm, −1 μm), (−1 μm, −1 μm) in the quaternion MFA, and letting (Δ*x*, Δ*y*) = (0, 0), (−1 μm, 1 μm), (1 μm, 1 μm), (1 μm, −1 μm), (−1 μm, −1 μm) in the quintuplet MFA. The laser power is 130 mW and the exposure times are, respectively, 10 s, 12 s, and 15 s for the triple, quaternion, and quintuplet MFA lithographing. Finally, the micro-nano structure arrays fabricated by the MFAs are shown in Figure 8(a1–c1), respectively.

Here, some quality factors are introduced to define the uniformity of focus intensity, positioning accuracy, and fabrication morphology [27].

(13) $$\left\{ \begin{array}{l} Q_{I}=1-\left(I_{\max}-I_{\min}\right)/\left(I_{\max}+I_{\min}\right)\\ Q_{D}=1-\left(D_{\max}-D_{\min}\right)/\left(D_{\max}+D_{\min}\right)\\ Q_{W}=1-\left(W_{\max}-W_{\min}\right)/\left(W_{\max}+W_{\min}\right) \end{array}\right.$$

where *Q_I_*, *Q_D_*, *Q_W_* are the quality factors for intensity, positioning accuracy, and fabrication morphology. *I*_max_, *I*_min_ denotes the maximum and minimum intensity of the focal spots in the MFAs, respectively. *D*_max_ and *D*_min_, respectively, refer to the maximum and minimum deviation of the structures from the desired position. *W*_max_ and *W*_min_ are the maximum and minimum line widths of the fabricated structure, respectively. When the *Q* factor equals 1, it indicates a good consistency of intensity, high positioning accuracy, and good fabrication morphology. From Figure 8d, it is seen that, the *Q_I_* is greater than 0.98 during production of the MFAs, theoretically. The positioning accuracy *Q_D_* is greater than 0.96, as shown in Figure 8e. We notice the micro-nano structures are slightly transformed; therefore, the line width is measured along the blue and red lines in Figure 8(a1–c1). The variation in *Q_W_* is shown as Figure 8f; the *Q_W_* value is greater than 0.92, generally. 

## 4. Conclusions

A noniterative phase segmentation, namely CSPS, was proposed to implement MFAs with controllable focus numbers, position, and morphology. The theoretical model was built up and the mechanism of CSPS was explained in detail. Based on the numerical simulation, the performance of the CSPS was investigated and discussed. The simulation results showed that it was convenient to manipulate MFAs by simply configuring the positional and morphologic parameter in the phase factor. Furthermore, the parallelized laser fabricating of micro-nano structure was implemented and investigated based on CSPS. The quality factors indicated that the positioning accuracy and the morphologic uniformity in the fabrication were greater than 96% and 92%, respectively. The CSPS could be a promising method of producing flexible MFAs for laser micro-nano fabricating, imaging, optical tweezers, and other fields.

## Data Availability

Not applicable.
